# Toxicokinetics of mercury in blood compartments and hair of fish-fed sled dogs

**DOI:** 10.1186/1751-0147-53-66

**Published:** 2011-12-07

**Authors:** Camilla L Lieske, Sara K Moses, Judith M Castellini, Jessica Klejka, Karsten Hueffer, Todd M O'Hara

**Affiliations:** 1 University of Alaska Fairbanks, Institute of Arctic Biology, 902 N Koyukuk Dr, Fairbanks, AK, 99775-7000, USA

**Keywords:** mercury, piscivore, canine, toxicokinetics, hair-excretion, hair to blood ratio

## Abstract

**Background:**

Understanding mercury (Hg) distribution in blood and the importance of hair as an excretory pathway is critical for evaluating risk from long term dietary Hg exposure. The major objective of this study was to characterize changes in total Hg concentrations in specific blood compartments and hair over time due to long term piscivory.

**Methods:**

Eight sled dogs (*Canis lupus familiaris*) were fed either a fish and kibble diet (n = 4), or a fish-free control diet (n = 4) for 12 weeks. Concentrations of Hg were monitored throughout the exposure period, and for 10 weeks post exposure, until Hg concentrations in all blood compartments of one of the exposed dogs dropped below detection limit. Additionally, foreleg hair was sampled during acclimation and weeks 0 and 12.

**Results:**

Hg was detected primarily in whole blood and packed cells, although it was sporadically detected at low concentrations in plasma and serum in two of the fish fed dogs. Dogs ingested an estimated average of 13.4 ± 0.58 μg Hg per kg body weight per day. Hg was detectable in whole blood and packed cells within a week of exposure. Detected concentrations continued to rise until plateauing at approximately 3-6 weeks of exposure at a mean of 9.2 ± 1.97 ng/g (ppb) in whole blood. Hg concentration decreased post exposure following 1st order elimination. The mean half-life (t_1/2_) in whole blood for Hg was 7 weeks. Mean Hg in hair for the fish-fed dogs at week 12 was 540 ± 111 ppb and was significantly greater (about 7-fold) than the Hg hair concentration for the control dogs. The hair to blood ratio for Hg in fish-fed dogs was 59.0 ± 7.6:1.

**Conclusions:**

This study found the sled dog model to be an effective method for investigating and characterizing blood Hg distribution (whole blood, serum, plasma, packed cells) and toxicokinetics associated with a piscivorous diet, especially for Hg-exposed fur bearing mammals (such as polar bears). Although hair excretion and hair to blood Hg ratios were not similar to human concentrations and ratios, the sled dog toxicokinetics of Hg in blood, was more similar to that of humans than traditional laboratory animals (such as the rat).

## Background

Conflicting studies have been published either extolling the benefits of a fish diet or cautioning against the risks of mercury (Hg) and other contaminants exposure [1-6]. Because of the importance of a marine based diet in many populations, both human and wildlife, it is critical to understand how Hg is absorbed, distributed, and eliminated in association with piscivory.

Many communities within Alaska rely on fish and other biota for subsistence. Sled dogs frequently live in close proximity to humans and consume the same diet. Published fish consumption advice for Alaskans developed by the Alaska Scientific Advisory Committee for Fish Consumption recommend an acceptable daily intake level (ADIL) of Hg, based on consumer body weight (BW), of 0.4 μg/kg BW/day [7]. This is intermediate to the Health Canada fish consumption recommendations of 0.2 μg/kg BW/day for children and women of childbearing age, and 0.5 μg/kg BW/day for everyone else [8]. In Alaska, recommended consumption allowances have been developed for the commonly eaten fish species for the state. The amount recommended depends on the amount of Hg generally detected in that species (or size) of fish. The limit for Alaskan salmon species is listed as unlimited.

Sled dogs have potential as biomonitors and models of species with a piscivorous diet. Sled dogs have been used as a surrogate to study organohalogen contaminants in top arctic predators (e.g. polar bears and arctic foxes) in Greenland [9-12], and have been found to be effective in providing direct evidence for cause and effect relationships with exposure. Hair from Alaskan sled dogs has been proposed for monitoring of Hg exposure [13] due to the similarity of their diet to a rural Alaskan subsistence diet. We propose utilizing sled dogs as a model of chronic exposure to Hg through piscivory. This model can be used to better understand toxicokinetics, toxicodynamics, relationships with chemical feeding ecology measures, genomics, etc.

Several studies have described the tissue distribution (concentrations and/or burdens) of Hg at a single time point [14,15] and others have described the toxicology of a large Hg exposure [16,17]. Studies describing the kinetics of Hg in blood have been done in laboratory rodents [18-20], fish [21], birds [22], and primates [23]. The rat model, in conjunction with data from monitoring Hg levels in human urine, feces, hair and blood over time have been used to develop a theoretical toxicokinetic model for predicting Hg distribution and elimination [24], however there are significant differences in the kinetics of Hg between rats and humans. Additionally, many of the laboratory kinetic studies (rodent, primate and avian) focus on exposure (intravenous or oral) of methylmercuric chloride rather than exposure to Hg from fish consumption.

Currently there is little information on the kinetics of Hg in piscivores, especially at relatively low concentration exposures. In order to better understand how blood Hg relates to marine fish intake, captive canids (sled dogs) on a known marine diet were sampled over time. Hair was sampled at different time points to evaluate its use in biomonitoring. We propose sled dogs as a model for understanding the blood compartmentalization of a low, chronic exposure to Hg. This paper describes the toxicokinetics of Hg in blood, including partitioning in whole blood (WB), serum, plasma and packed cells from serum and plasma preparations (PCserum and PCplasma).

## Methods

### Animals

The study animals were eight (four female and four male) mixed breed sled dogs (*Canis lupus familiaris*). Ages ranged from 3 to 8 years. Dogs were randomly assigned feeding regiments (fish-fed or kibble only) and kept in outside pens separated according to feeding groups. The dogs had access to water *ad lib *during the study period. Animal handling and the feeding regime were performed according to protocols approved by the University of Alaska Fairbanks Institutional Animal Care and Use Committee (protocol #145639-2). Whole blood sampled two weeks prior to the start of the fish exposure and at week 12 of the study (the end of the fish exposure) was submitted to Veterinary Services at the University of Alaska Fairbanks for determination of clinical chemistry, hematology and evaluation of health. All dogs were found to be within expected ranges for the health parameters.

### Diet

The control group (n = 4, 2 males and 2 females) was fed commercial dog kibble (Standard Choice 26% Value Meal Dog Food, Fromm Family Foods, Mequon, WI) twice daily throughout the entire study. The fish-fed group (n = 4, 2 males referred to as dogs K and T; and 2 females referred to as dogs C and M) received a 50% kibble, 50% fish diet (the same kibble as the control group for one meal and keta (chum) salmon (*Oncorhynchus keta*) for the other meal) to result in similar energy (caloric) intake as for the controls. The salmon was provided frozen (Interior Alaska Fish Processors, Fairbanks, AK) and cut frozen into pieces of approximately 300 g (range:198-458 g) prior to the start of the study. Sections (5 from the cranial end, 5 from the caudal end) from 10 different fish were analyzed at the beginning of the study for Hg to determine the range of Hg concentrations in the salmon fed to the dogs. These concentrations along with mass of the fish fed were used to estimate daily intake of Hg. Fish meals were packaged individually and heated in a microwave until any liquid in the package was brought to a boil to inactivate potential parasites and any thiaminase present. Packaged fish meals remained frozen (-20°C) until feeding. Dogs were fed individually to control the amount of food intake per dog and the fish amount recorded for each daily feeding. The amount fed was adjusted as needed to maintain body condition and BW as determined by the primary feeder from 3 May - 26 October 2010. BW was measured at each sampling event.

### Study Design

The study was carried out in three phases: acclimation to kibble diet, exposure to fish meals and fish-free (kibble-only) meals, and elimination of Hg phase. These phases consisted of the following: During a three-week period (3-23 May 2010), all eight dogs were on the control (fish meal-free kibble) diet. After this acclimation phase, 4 dogs (fish-fed group) were switched to a 50% marine fish, 50% kibble (one fish meal and one kibble meal per day) diet based on mass of the dog and calories required for 12 weeks (24 May-15 Aug 2010). All dogs were fed the commercial kibble diet (no fish) during the 10 week Hg elimination phase (16 August-26 October 2010).

### Blood

Blood was drawn from the cephalic vein using a 21 gauge × 3/4" Vacutainer blood collection set (Becton, Dickinson & Co, Franklin Lakes, NJ) and sampled directly into 6 ml Vacutainer Trace Element Tubes (one serum (with no additive) and one K_2_EDTA tube (Becton, Dickinson & Co, Franklin Lakes, JN)). Blood was sampled 2 weeks prior to starting the fish exposure, weekly from weeks 0-4 during the fish exposure, then every second week for weeks 6-12 of the exposure phase and the 10 week elimination phase (weeks 13-22). Hematocrit (Packed cell volume (PCV)) was determined during the acclimation phase and for weeks 2-22 of the study using the whole blood from the K_2_EDTA tube drawn into a micro-hematocrit capillary tube (Fisher, Pittsburgh, PA) and centrifuged (3 minutes MCHT setting, 10,400 RPM, 12,600 *g*) using a Clay Adams Triac Centrifuge (Becton, Dickinson & Co, Franklin Lakes, NJ). An additional 1-1.5 ml aliquot of whole blood was placed in a cryovial (Nalgene, Rochester, NY) for later Hg analysis, and the remaining whole blood was centrifuged (5 minutes blood separation setting, 3500 RPM, 1500 *g*) using the Clay Adams Triac Centrifuge separated into plasma and packed cells (PCplasma). Blood from the serum tube was similarly centrifuged and separated into serum and packed cells (PCserum). Whole blood and the various blood compartments were kept frozen at -20°C until analyzed.

### Hair

Hair samples (a mixture of guard hairs and undercoat) were collected using an electronic hair clipper from each dog in the study three times: two weeks prior to being fed fish, the first day of week 0 and on the last day of the exposure (week 12). Hair samples were from over each animal's cephalic vein (foreleg hair). Hair was washed using a 1% Triton-X 100 (EMD Serono, inc, Rockland MA, USA) soap solution, rinsed thoroughly and freeze dried using a Freezone 4.5 Freeze Dry System (Labconco, Kansas City, MO, USA).

### Mercury analysis

Hg was determined using a DMA-80 direct Hg analyzer (Milestone, Sorisole, BG, Italy). Instruments were calibrated using a 6 point linear calibration curve from 0.25 ng to 7.00 ng. Samples were considered below detection limit (BDL) when the Hg detected in the sample was below 0.25 ng for at least 2 of the repeated samples. Approximately 125-150 mg of blood or 10-20 mg of hair was analyzed at a time. All samples were analyzed in duplicate. A third sample was analyzed when results of the duplicates differed by more than 10% (blood) or when sample abundance permitted (hair). At the beginning of each run, blanks, aqueous standards of known Hg concentrations, and a certified reference material (DORM3 (National Research Council Canada, Ottawa ON, Canada) for runs with blood samples, IAEA-086 Human Hair (International Atomic Energy Agency Analytical Quality Control Services, Vienna, Austria) for runs with hair) were analyzed for quality assurance and control. Blanks were below 0.10 ng, Hg, standards were all within 10% of expected values, and the total Hg concentration in the reference material was within the certified range.

### Calculations and Statistics

For samples that were above detection limit (> 0.25 ng of Hg), differences among exposed dogs for blood matrices (whole blood, PCplasma, PCserum) were determined using a non-parametric Friedman test with all pairwise comparisons done using a Conover test [25]. Hg concentration in whole blood was adjusted for PCV and weight before comparisons. Time to Hg plateau for each matrix was determined using Scheffé multiple comparison identifying the earliest week not found to be significantly different from week 12. Elimination was calculated assuming a two compartment open model with 1^st ^order elimination using the equation

$$d\text{Hg}/d{\text{t=-k}}_{\text{e}}\text{Hg}$$

The k_e _is the elimination rate constant expressed as week^-1 ^Half-life (t_1/2_) can be calculated as t_1/2 _= 0.693/k_e _[26]. Parameters (time to plateau, k_e_, t_1/2_) for individuals (dogs C, K, M and T) were calculated separately as well as calculated using the combined results. Two models for 0 order (constant rate) elimination were also fit to the data for comparison of mean square error. Rate constants were calculated using the slope from week 12 (end of exposure) and week 22 (end of study) and a constant 1% loss due to red blood cell (RBC) turnover.

For each time period, differences between hair Hg for controls and fish-fed dogs were determined using a Kruskel-Wallis Test. Difference within each treatment (control or fish-fed) were evaluated using a Friedman test blocked by dog.

Calculations were performed using Microsoft Office Excel 2003 (Microsoft Corporation, USA) and statistical comparisons were done using StatsDirect version 2.7.8 (Altrincham, Cheshire, UK). An α < 0.05 was considered significant for all comparisons.

## Results

### Exposure Estimation and Detection of Blood Mercury

**Figure 1:**
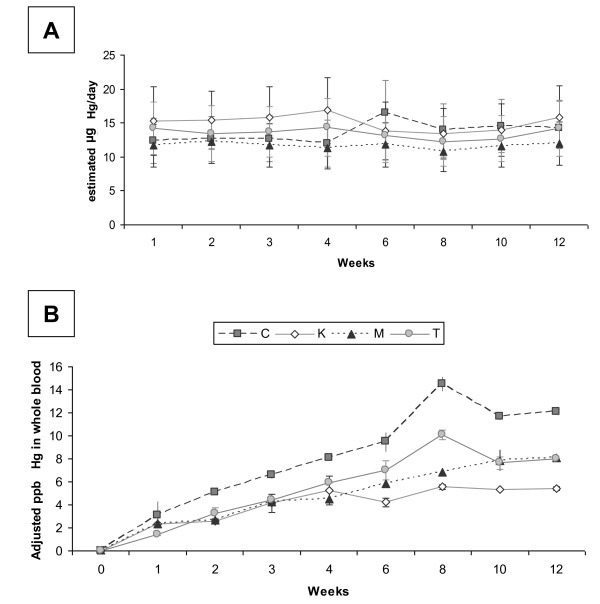
**Mercury intake and blood total mercury levels**. A) Estimated daily total mercury (Hg) exposure from fish for the 4 dogs on the fish diet B) Hg (corrected for BW and PCV differences amongst the dogs) detected in whole blood over the 12 weeks of exposure for the 4 dogs on the fish diet.

**Figure 2:**
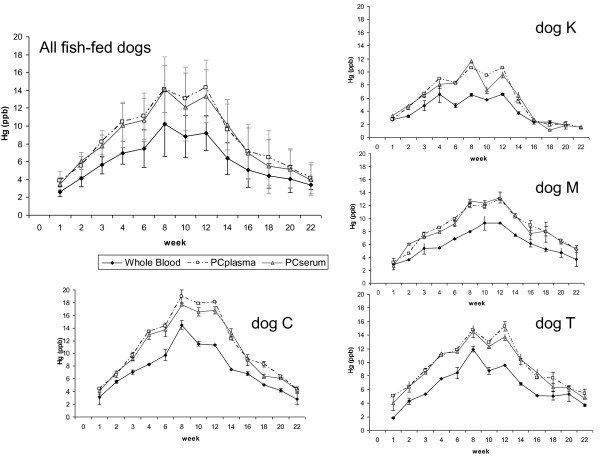
**Mercury in blood compartments**. Mercury (Hg) detected in each blood compartment for each individual dog and all fish-fed dogs combined. Error bars represent differences in results amongst the repeated analysis for each sample.

The Hg concentration mean ± SD of the chum salmon was 45.5 ± 11.8 ppb. Fish diet dogs (dogs C, K, M, T) were fed between 11.4 and 18.9 g fish/kg BW per day (average of 13.4 ± 0.58 μg Hg per kg BW per day (Table 1), with variation to maintain BW and condition. The amount fed was used to estimate Hg exposure (Figure 1A). Hg was detectable in whole blood, PCplasma, and PCserum within 1 week after exposure (Figures 1B and 2) and plateaued within 3-8 weeks (average = 3-6 weeks) (Table 2). During the exposure phase, Hg was detected just above the detection limit in plasma for dog K at week 3 and in both plasma and serum for dog C in week 8. All other plasma and serum sample time points were below detection limit. Hg was below detection limit for all matrices analyzed from the control dogs.

**Table 1:** Amount fish fed.

	dog C		dog K		dog M		dog T	
	g fish per meal	g fish per kg BW	g fish per meal	g fish per kg BW	g fish per meal	g fish per kg BW	g fish per meal	g fish per kg BW
week	*Mean ± SD*	*Mean ± SD*	*Mean ± SD*	*Mean ± SD*	*Mean ± SD*	*Mean ± SD*	*Mean ± SD*	*Mean ± SD*
**1**	268 ± 23.5	15.0 ± 1.31	343 ± 69.2	16.4 ± 3.31	257 ± 17.4	12.0 ± 0.82	314 ± 22.7	13.8 ± 1.00

**2**	267 ± 11.8	14.1 ± 0.62	322 ± 30.1	14.9 ± 1.39	255 ± 10.9	11.4 ± 0.48	295 ± 46.3	13.0 ± 2.04

**3**	289 ± 21.5	15.3 ± 1.14	359 ± 44.2	16.3 ± 2.01	270 ± 21.2	12.0 ± 0.94	311 ± 26.5	14.1 ± 1.20

**4**	266 ± 31.4	14.8 ± 1.75	362 ± 44.1	16.6 ± 2.02	256 ± 17.4	12.4 ± 0.84	306 ± 45.5	13.9 ± 2.06

**6**	349 ± 48.4	18.9 ± 2.63	311 ± 50.0	14.2 ± 2.29	259 ± 24.2	13.0 ± 1.21	298 ± 43.5	13.5 ± 1.97

**8**	322 ± 36.8	17.7 ± 2.02	294 ± 29.5	13.8 ± 1.38	241 ± 22.0	12.2 ± 1.11	273 ± 41.0	12.9 ± 1.94

**10**	319 ± 24.5	18.5 ± 1.42	313 ± 31.3	15.0 ± 1.50	257 ± 14.5	13.3 ± 0.75	282 ± 20.5	13.6 ± 0.99

**12**	312 ± 38.9	18.1 ± 2.25	338 ± 43.9	16.0 ± 2.08	261 ± 13.9	13.5 ± 0.72	304 ± 38.3	14.4 ± 1.81

Average grams fish fed per meal and per kg dog weight during exposure. Variation in grams fish fed per kg body weight (BW) was due to the effort to maintain BW and condition by adjusting meal sizes as needed.

**Table 2:** Summary of toxicokinetic variables in various compartments (blood and hair).

	Compartment	Hg at week 12 (ppb)	Time to Plateau^a ^(weeks)	k_e_^b^ (weeks^-1^)	t_1/2_ (weeks)	Ratio Hg Hair:Blood^c^
**dog C**	**WB:**	11.36	8	-0.14	-5.0	62
	**PCp:**	18.98	8	-0.14	-4.9	
	**PCs:**	16.80	6	-0.14	-5.1	
	**Hair:**	704.9				

**dog K**	**WB:**	6.58	2	-0.16	-4.3	69
	**PCp:**	10.64	4	-0.21	-3.3	
	**PCs:**	9.60	3	-0.21	-3.4	
	**Hair:**	456.6				

**dog M**	**WB:**	9.27	6	-0.09	-7.5	52
	**PCp:**	12.99	8	-0.09	-7.7	
	**PCs:**	13.24	8	-0.09	-7.6	
	**Hair:**	488.1				

**dog T**	**WB:**	9.57	4	-0.10	-7.3	54
	**PCp:**	15.28	6	-0.11	-6.6	
	**WB:**	9.57	4	-0.10	-7.3	54
	**PCp:**	15.28	6	-0.11	-6.6	
	**PCs:**	13.71	4	-0.11	-6.6	
	**Hair:**	526.0				

**Average^d^**	**WB:**	9.2 ± 1.97	3	-0.10	-7.0	59 ± 7.6
**all fish-**	**PCp:**	14.2 ± 3.18	6	-0.11	-6.6	
**fed dogs**	**PCs:**	13.3 ± 2.95	6	-0.10	-6.9	
	**Hair:**	544 ± 111.0				

Total mercury (Hg) concentration was determined at the end of fish exposure (week 12) for the fish-fed dogs and the rates to plateau and elimination for the different blood compartments (whole blood (WB), packed cells from plasma (PCp) and packed cells from serum (PCs)) were calculated.

^a^Time to plateau was determined as the earliest time point found not to vary significantly from Hg concentration at week 12 (α = 0.05).

^b^k_e _is the first order elimination constant

^c^Ratio = Hg Hair (ppm)/Hg Whole Blood (ppm)

^d^Analysis was done on the average of the combined parameter results

Within each exposed dog, Hg in PCserum and PCplasma did not vary significantly from each other. Hg in whole blood varied significantly from both PCserum and PCplasma (*P *< 0.0001 for dogs C, M, T and *P *= 0.0006 and *P *= 0.0002 for PCserum and PCplasma, respectively, for dog K). For each blood compartment (whole blood, PCserum and PCplasma) all dogs varied significantly from each other (*P *ranged from 0.0339 to < 0.0001) with the exception of dogs M and T for whole blood (*P *> 0.99) and PCserum (*P *= 0.35). For all dogs and all weeks, PCV ranged from 45.5 to 56.5%. Within each dog across all weeks the average PCV was 51 ± 3%).

### Elimination

**Figure 3:**
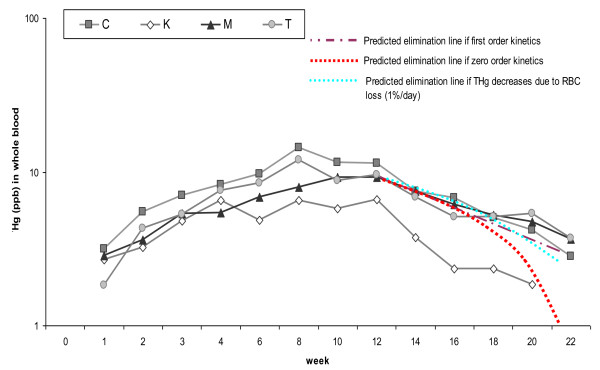
**Mercury in whole blood**. Semi-log graph of the mercury (Hg) in whole blood over entire time course of the experiment. Three model prediction lines have been added. The predicted elimination line for 1^st ^order kinetics and zero order elimination were based on the average k_e _(1^st ^order tem) or k_0 _(zero order term) for all fish-fed dogs. The third prediction line assumes the elimination of Hg is due to the turnover of red blood cells (estimated at 1% per day).

All fish-fed dogs appeared to undergo first order elimination (Figure 3). The elimination half-life in whole blood for individual dogs ranged from 4.3 to 7.5 weeks (30 to 52.5 days), while the average for all fish-fed dogs was 7 weeks (49 days) (Table 2). Only dog K dropped below detection limit in all matrices before the end of the study. For all fish-fed dogs, the mean square error was lowest for the 1^st ^order elimination model. The mean square error for the 1^st ^order elimination model ranged from 2-15 times lower than the 0-order model using the linear slope (average of 6 ± 5 times lower) and 2-7 times lower for the 0-order model using the 1% RBC turnover model (average 3 ± 2.1 times lower).

### Mercury in hair

**Figure 4:**
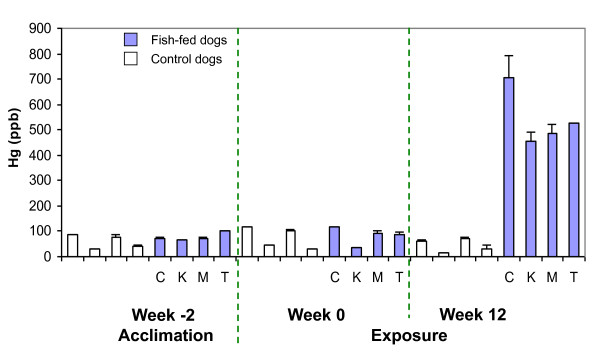
**Mercury in hair**. Comparison of mercury (Hg) in hair between control and fish-fed dogs. Error bars represent the difference between repeated measures for each sample.

There was no significant difference in hair Hg between control and fish-fed dogs for weeks -2 (acclimation) and week 0. At week 12 (end of exposure) there was a significant (*P *= 0.02) increase in hair Hg between control and fish-fed dogs (Figure 4). Comparing results for each dog across all weeks evaluated, there was no significant difference in the control dogs (*P *= 0.18), but there was a significant difference between the fish-fed dogs at week 12 compared to either week 0 or -2 (*P *= 0.01). Hg in hair at week 12 was 6, 13, 5, and 6 times greater than for weeks 0 and -2 for dogs C, K, M, and T respectively. The ratio of Hg in hair to whole blood ranged from 54 to 69 (Table 2).

## Discussion

We found that Hg from ingested fish increased to detectable concentrations in certain blood compartments within a week of exposure. Although there were differences in time to uptake and elimination amongst the chronically exposed dogs, in general, the patterns of uptake and elimination of Hg were similar. In addition, the rates of elimination determined for the dogs in this study were consistent with those predicted in toxicokinetic models for human exposure [24]. Because of this consistency, the sled dog model can be a powerful tool in better understanding the toxicology and toxicokinetics of Hg in piscivores, and warrants further detailed study, especially with respect to subarctic and arctic food webs.

Hg is compartmentalized primarily in the RBCs (packed cells), most likely binding to the sulfhydryl groups of hemoglobin and other proteins [27]. This is consistent with other studies that have assessed Hg in blood [14,21,28]. The lack of significance between PCserum and PCplasma within each individual dog supports the hypothesis that the Hg is carried primarily on or in the RBC fraction, and not detectably bound to other proteins in the serum or plasma at this level of Hg intake from fish. Future studies may consider analyzing only one or the other of the packed cell fractions, rather than both. Because the PCVs for the dogs in this study were relatively consistent, PCV did not significantly affect the comparisons. However, because of the primary compartmentalization of Hg in RBCs and the potential importance of PCV on Hg concentration in whole blood it is critical to also evaluate PCV. The sporadic detection of Hg in serum and plasma for dogs K and C may be related to the slightly greater amount of fish these dogs were fed. It is possible that with a diet higher in Hg, an increased detection of Hg in plasma and serum may occur. Based on other studies, this compartment (serum or plasma) would likely have a very different k_e _and t_1/2 _compared to whole blood, PCplasma or PCserum assessed in this study [20,21]. This study represents exposure to a diet with a relatively low, but biologically and ecologically relevant, Hg concentration. Calculated rates may change with exposure to higher concentrations of Hg, so this study should not be extrapolated as a model for exposure to high levels of dietary Hg.

Based on this study, relatively low circulating concentrations of Hg appears to undergo 1^st ^order elimination. Prior research has determined that canine RBC turnover is around 1% per day (for a RBC lifespan of 100-115 days) [29]. If the primary route of elimination for Hg were through RBC turnover, we would expect the elimination curve to have more closely followed the zero-order elimination curves shown in Figure 3. The 1% RBC turnover model does fit the data better than the average linear slope 0-order model. This is likely due to the compartmentalization of Hg in RBCs and the contribution of RBC turnover to Hg elimination. Although the 1% RBC turnover model may provide an approximation of elimination rates when k_e _is unknown and 1^st ^order kinetics cannot be calculated, use of alternative models (such as the 1% RBC turnover model) would result in underestimating predicted blood Hg concentrations at any given time, in addition to underestimating the time for elimination of Hg from blood.

Our canine model not only is of use in modeling canine Hg exposure, but it is also a good model for other piscivores including humans. The rate of elimination of Hg from blood determined in this study is more similar to that predicted by human models of toxicokinetics than the laboratory rodent models have found (49 days in this study, 52 days for humans [24], and 5.6-9.3 days in mice [20]). The estimated Hg intake level for the dogs in our study was 0.80 μg/kg BW/day and was the equivalent of a 70 kg person eating 1 kg of fish a day. Although this is about twice the Alaska ADIL for humans, blood Hg in the dogs plateaued at 8-16 ppb which is below the 20 ppb level Health Canada has applied as their limit for "normal" in their biomonitoring program [8].

The differences noted among the dogs, most notably dog K (more rapid time to plateau, faster rate of elimination) may indicate genetic differences in Hg kinetics. In humans, differences in glutathione expression can significantly impact Hg elimination [30,31]. Whether the relationship between gene expression and Hg disposition is also true for canines has not been described. It is interesting that the dog K had the highest hair Hg to blood Hg ratio. It is possible that the more rapid elimination noted was due to sequestration in hair.

Hair proved to be a good indicator of exposure to Hg via a fish diet. In other mammals a linear relationship between hair and blood has been seen [32], and in humans it is used for risk assessments to predict blood Hg from hair concentrations [33]. In harbor seals the ratio of Hg in hair to Hg in blood is a bit lower (22 in females, 40 in males) [32] than was found in the sled dogs (average of about 59). In polar bears the ratio appears to be a bit higher than in the sled dogs (estimated at about 100× based on average blood and hair Hg) [34]. However, for both these species the hair to blood ratio was much closer to the ratio found in this study than the ratio seen in humans (Hg averages about 250× whole blood Hg concentration [33]). The differences may be due to the relative surface area and hair density in dogs, seals, polar bears and humans. Because Hg is primarily detected in RBCs, PCV may also be an important factor in differences seen.

The concentration of Hg detected in sled dog hair in this study (all below 1 ppm) was well below the concentration associated with toxicity in wildlife [35]. Subclinical neurological effects have been measured in polar bear brains at Hg concentrations associated with 6 ppm in hair [35], and clinical neurological effects in mink at Hg concentrations over 30 ppm in hair [36]. Although this study did not directly assess health effects on the sled dogs associated with exposure to Hg (such as renal damage, changes in thyroid hormones, and neurological damage [11,37-39]), it does demonstrate that sled dogs are an effective model for Hg exposure, and can be used to design future studies assessing health effects.

## Conclusions

Overall, the sled dog model provides an effective surrogate for piscivorous species. Blood elimination rates are more similar to human rates than the more commonly used mouse or rat models. Additionally, hair Hg is an effective means of monitoring exposure to Hg via diet. Hair to blood ratios were found to be more similar to other mammalian species than humans, and could be useful in assessing risk to other species such as piscivorous wolves or marine mammals. This study evaluates Hg exposure in a manner that is reflective of populations (human or wildlife) relying on fish for subsistence as is still common in many areas of the circumpolar North.

## Abbreviations

Hg: Total mercury; ADIL: acceptable daily intake level; BW: body weight; RBC: red blood cell; PCplasma: packed RBCs from plasma; PCserum: packed RBCs from serum; WB: whole blood; t1/2: half-life; k_e_: 1^st ^order elimination constant.

## Competing interests

The authors declare that they have no competing interests.

## Authors' contributions

CLL participated in the study design and coordination, assisted with sample collection and Hg analysis, completed the statistical analysis and drafted the manuscript. SKM and JMC participated in sample collection and Hg analysis. JK prepared and analyzed the hair for Hg and preformed initial statistical analysis on the results. KH participated in the study design, fish exposure, and sample collection. TMO conceived of the study, participated in its design and coordination, in addition to sample collection. All authors read and approved the final manuscript.
